# The relationship between Subfoveal Choroidal Thickness and Hypertensive Retinopathy

**DOI:** 10.1038/s41598-021-84947-7

**Published:** 2021-03-09

**Authors:** Lei Shao, Ling Xiao Zhou, Liang Xu, Wen Bin Wei

**Affiliations:** 1 Beijing Tongren Eye Center, Beijing Key Laboratory of Intraocular Tumor Diagnosis and Treatment, Beijing Tongren Hospital, Capital Medical University, 1 Dong Jiao Min Xiang, Dong Cheng District, Beijing, 100730 China; 2 The First Affilitated Hospital of Xi’an Medical University, Xi’an, China; 3 Beijing Institute of Ophthalmology, Beijing Tongren Hospital, Capital Medical University, Beijing, China

**Keywords:** Epidemiology, Retinal diseases

## Abstract

The Beijing Eye Study 2011 is a population-based cross-sectional study in Northern China, which enrolled 3468 participants whose age were more than 50 years. A detailed ophthalmic examination was performed including spectral-domain optical coherence tomography with enhanced depth imaging for measurement of SFCT and fundus photography. Blood pressure, fundus photographs and choroidal OCT-images were available for 3237 (93.3%) subjects, with 1953 (56.3 ± 0.8%) of the study population fulfilled the diagnosis of hypertension and 1089 subjects having hypertensive retinopathy. For the hypertensive cases, the SFCT in patients with hypertensive retinopathy (286.48 ± 105.23 µm) was significantly thicker than subjects without hypertensive retinopathy (187.04 ± 78.80 µm, P < 0.001). SFCT was significantly associated with the stage of hypertensive retinopathy (P < 0.001), but not significantly associated with diastolic blood pressure (P = 0.94), history (P = 0.95) and years (P = 0.91) of hypertension. In conclusion, hypertension as systemic disease was not significantly affect the subfoveal choroidal thickness, but as ocular disease, hypertensive retinopathy was significantly related to changes of choroidal thickness. Lesions of choroid during chronic hypertension may play an important role in development of hypertensive retinopathy.

## Introduction

Hypertension is often asymptomatic and recording blood pressure is opportunistic. Although the prevalence varies by ethnic group and country, which ranges from 26.6% to 35.5%, hypertension was very common all over the world^1–3^. A number of hypertensives may present for the first time with target-organ damage involving various organs^2–4^. Hypertension can cause not only cute ocular abnormalities but also chronic ocular abnormalities, such as choroidopathy, retinopathy, and optic neuropathy^5^. Hypertensive retinopathy is the microvascular abnormalities in hypertensive patients, which can be divided into three phases, including systolic, sclerotic, and exudative phases. These three phases do not necessarily in this sequence. Choroid ischemia or choroidal hyperperfusion maybe play an important role in the pathogenesis of hypertensive chorioretinopathy, which develops because the automatic regulation ability of retinal vasculature cannot control the choroid vasculature^5–7^. Since hypertension was probably associated with lesions of retina and choroid, understanding the choroidal vascular abnormalities which resulted from elevation of blood pressure seems to be important. Because the choroid receives about 95% of the ophthalmic artery blood, which provide oxygen and supply nutrition to the outer retinal layers.

After the landmark study by Spaide and colleagues who described the technical possibility to visualize the choroid and measure its thickness by using the enhanced depth imaging mode of spectral-domain optical coherence tomography (EDI SD-OCT)^8^, several studies using this method have report the subfoveal choroidal thickness (SFCT) in normal subjects and patients with choroidal or retinal diseases^9–12^. Because the referral bias in hospital-based study is inevitable, our study measured the subfoveal choroidal thickness in a population-based research which recruit a relatively study sample; and reveal that whether hypertension as systemic disease, and hypertensive retinopathy as ocular disease, especially different stages of hypertensive retinopathy is associated with an abnormal thickness of the subfoveal choroid.

## Methods

### Study design and patients

The Beijing Eye Study 2011 was a population-based cross-sectional in Northern China^13^. The protocol was approved by Medical Ethics Committee of the Beijing Tongren Hospital, and experiment was conducted in accordance with the relevant guidelines and regulations. All participants signed the informed consent voluntarily. 5 urban communities and 3 rural communities of Beijing were recruited in the cluster sampling. The only inclusion criterion is an age of 50 +, and out of an eligible population of 4403 individuals, 3468 (78.8%) individuals [1963 (56.6%) women] took part in our study. 1633 (47.1%) participants [943 (57.7%) women] came from the rural part, and 1835 (52.9%) participants [1020 (55.6%) women] came from the urban part. The mean age of the population was 64.6 ± 9.8 years (median, 64 years; range, 50–93 years).

After getting an informed consent, all study participants underwent an interview by using a standard questionnaire about their family and personal information. Then they went for fasting blood test of blood lipids, glucose and glycosylated hemoglobin HbA1c. Blood pressure, body height and weight and the circumference of the waist and hip were recorded. At last, a detail ophthalmic examination was preformed including spectral domain optical coherence tomography (SD-OCT; Spectralis, Wavelength: 870 nm; Heidelberg Engineering Co., Heidelberg, Germany) with enhanced depth imaging (EDI) modality on the fovea and fundus photographs of central fundus after pupil dilation. All measuring items and methods can be seen in the references^12,13^.

### Classification of hypertensive retinopathy

**Figure 1:**
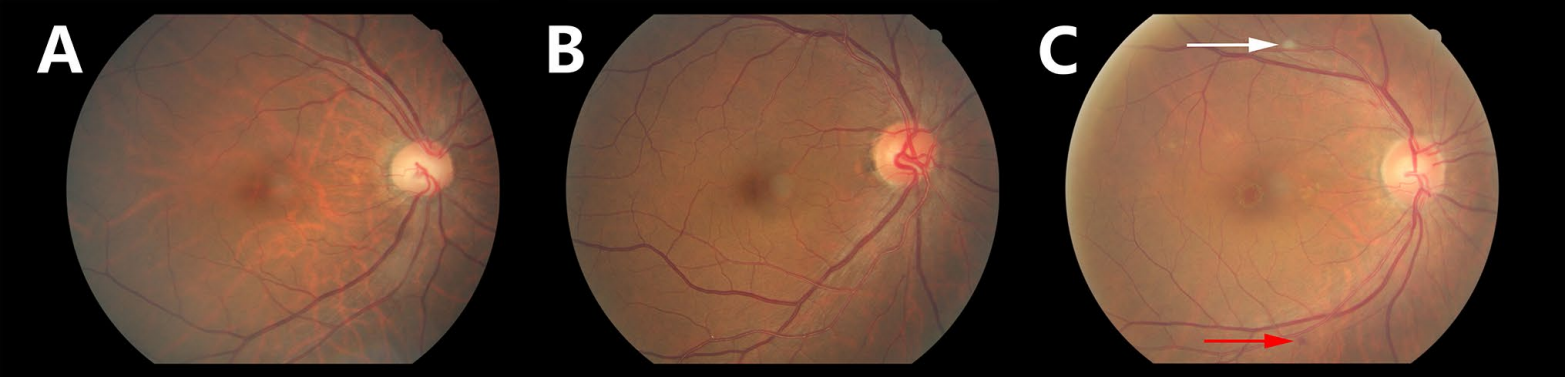
Classification of hypertensive retinopathy. (**A**) Mild hypertensive retinopathy (slight or modest narrowing of retinal arterioles with an arterial: venous ratio of more than 1:2). (**B**) Modest hypertensive retinopathy (modest to severe narrowing of retinal arterioles with an arterial: venous ratio less than 1:2 or arteriovenous nicking). (**C**) Severe hypertensive retinopathy (cotton wool spot and flame-shaped hemorrhages, or optic nerve edema); white arrow: cotton wool; red arrow: hemorrhage.

Masked manner was performed in evaluating the hypertensive retinopathy with fundus photographs (non-stereoscopic photograph of 45° of the central fundus; fundus camera type CR6-45NM, Canon Inc. USA). The diagnosis for each participant was according to the worse grade in both eyes. The classification was based on Keith, Wagerner, and Barker^6,14^. The severity of hypertensive retinopathy was graded into mild hypertensive retinopathy (slight or modest narrowing of retinal arterioles with an arterial: venous ratio of more than 1:2), modest hypertensive retinopathy (modest to severe narrowing of retinal arterioles with an arterial: venous ratio less than 1:2 or arteriovenous nicking), and severe hypertensive retinopathy (cotton wool spot and flame-shaped hemorrhages, or optic nerve edema) (Fig. 1).

The photographs were assessed by an experienced and trained ophthalmologist (LS). In case of doubt, the photographs were re-assessed by a panel including several ophthalmologists (LS, QSY, WBW). For study purposes, we diagnosed hypertension as systolic blood pressure (SBP) ≥ 140 mmHg, diastolic blood pressure (DBP) ≥ 90 mm Hg, or current treatment with antihypertensive drugs in subjects with a history of hypertension^15,16^.

### SFCT measurements

Subfoveal choroidal thickness (SFCT) was measured using a spectral domain optical coherence tomography (SD-OCT; Spectralis, Wavelength: 870 nm; Heidelberg Engineering Co., Heidelberg, Germany) with enhanced depth imaging (EDI) modality after pupil dilation. Seven sections, each comprising 100 averaged scans, were obtained in an angle of 5°–30° rectangle centered onto the fovea. The horizontal section running through the center of the fovea was selected for further analysis. Subfoveal choroidal thickness was defined as the vertical distance from the hyperreflective line of the Bruch’s membrane to the hyperreflective line of the inner surface of the sclera. The measurements were performed using the Heidelberg Eye Explorer software (version 5.3.3.0; Heidelberg Engineering Co., Heidelberg, Germany). Only the right eye of each study participant was assessed. The images were taken by one technician (CXC) and the images were assessed by two ophthalmologists (LS, KFD). The reproducibility of the technique was previously examined and revealed a high reproducibility (Bland–Altman plot with 1.9% (61/3233) points outside the 95% limits of agreement; intra-class coefficient: 1.00; mean coefficient of variation: 0.85% ± 1.48%)^17^.

### Statistical analysis

Statistical analysis was performed using a commercially available statistical software package (SPSS for Windows, version 20.0, IBM-SPSS, Chicago, IL). In a first step, we examined the mean values (presented as mean standard deviation) of SFCT. Frequencies were presented as mean ± standard error. In a second step, we performed a univariate linear regression analysis with SFCT as dependent parameter and ocular and general parameters as independent parameters. In a third step, we carried out a multivariate regression analysis using the stepwise method, with explanatory variables be appropriately selected (See the following steps for details). In a fourth step of the analysis, we added hypertension related parameters to the multivariate analysis, independently whether they were, or were not, significantly associated with SFCT in the univariate analysis. In a fifth step, we dropped step by step the hypertension related parameters from the list of independent variables, starting with the parameter with the highest P-value. In a second part of the statistical analysis, we assessed associations between hypertension, or hypertensive retinopathy, with systemic and ocular parameters. We then performed a multivariate regression analysis which included in its list of independent variables all parameters which were significantly associated with hypertension or hypertensive retinopathy in the univariate and then added SFCT as additional independent parameter. 95% confidence intervals (CI) were presented. All P-values were 2-sided and were considered statistically significant when the values were less than 0.05.

## Results

### Patient enrollment and characteristics

Out of the 3,468 subjects participants enrolled in the study, blood pressure, fundus images and OCT images of choroid were available for 3237 (93.3%) participants with a mean age of 64.26 ± 9.63 years (median: 63 years; range: 50–93 years), a mean refractive error of − 0.19 ± 2.11 diopters (median: 0.25 diopters; range: − 22.00 to + 13.50 diopters) and a mean axial length of 23.25 ± 1.13 mm (median: 23.13 mm; range: 18.96–30.88 mm). The subjects participating in the study as compared to the non-participating subjects were significantly younger (age: 69.62 ± 10.97 years; *P* < 0.001) and less myopic (refractive error: − 0.80 ± 3.62 diopters; *P* < 0.001). But the incidence of hypertensive retinopathy (*P* = 0.41) and axial length (*P* = 0.31) were not significantly different in paricipanting and non-participanting group.

Systolic blood pressure (SBP) ≥ 140 mmHg, or/and diastolic blood pressure (DBP) ≥ 90 mmHg were detected in 1010 (29.1%) subjects. Hypertension was known for 1568 (45.2%) individuals who had a diagnosis or treatment. Altogether, 1953 (56.3 ± 0.8% (95% CI 54.7, 57.7) of the study population fulfilled the diagnosis of hypertension. The mean age was 66.39 ± 9.80 years, the refractive error was − 0.17 ± 2.29 diopters, and man axial length was 23.20 ± 1.13 mm. The mean SFCT was 246.77 ± 107.22 µm (median: 244 µm; range: 8–724 µm). Out of the 1953 patients with hypertension, 1089 (55.76 ± 1.51%) subjects showed a hypertensive retinopathy.

### Change in SFCT

Mean SFCT of patients with hypertension (246.77 ± 107.22 µm (median: 244 µm; range: 8–724 µm) was significantly thinner than non-hypertensive subjects (261.91 ± 107.73 µm (median: 252 µm; range: 3–854 µm) *P* < 0.001). But for the hypertensive cases, the SFCT in patients with hypertensive retinopathy (286.48 ± 105.23 µm (median: 297 µm; range: 12–724 µm) was significantly thicker than subjects without hypertensive retinopathy (187.04 ± 78.80 µm (median: 187 µm; range: 8–456 µm); *P* < 0.001); and each grade of hypertensive retinopathy have a thicker SFCT than that without hypertensive retinopathy (Table 1). However, SFCT in different classification (grade1 to 3) of hypertensive retinopathy was no significant difference (*P* all > 0.05).

**Table 1:** SFCT in different classification of hypertensive retinopathy.

Classification of hypertensive retinopathy	N	SFCT (mean ± SD, µm)	Range (µm)	*P*
0	726	187.04 ± 78.80	8–456	
1	396	289.11 ± 100.87	12–570	< 0.001
2	559	286.10 ± 111.07	13–724	< 0.001
3	134	280.28 ± 92.39	53–513	< 0.001

### Univariate analysis of SFCT

Univariate analysis showed that the SFCT was significantly related to the following systemic parameters: younger age (*P* < 0.001), male gender (*P* = 0.006), greater body height (*P* < 0.001), weight (*P* < 0.001), rural region of habitation (*P* < 0.001), higher diastolic blood pressure (*P* < 0.001), history (*P* < 0.001) and years (*P* < 0.001) of arterial hypertension, higher serum concentrations of glucose (*P* = 0.011) and cholesterol (*P* = 0.029), smoking (*P* < 0.001), higher alcohol consumption (*P* < 0.001), less aspirin intake (*P* < 0.001), frequency of reported snoring (*P* = 0.011) and development of hypertensive retinopathy(*P* < 0.001); and related to the ocular parameters as following: higher best corrected visual acuity (*P* < 0.001), higher intraocular pressure (*P* < 0.001), shorter axial length (*P* < 0.001), hyperopic refractive error (*P* < 0.001), steeper cornea (*P* < 0.001), lower anterior chamber depth (*P* < 0.001), and thinner lens (*P* < 0.001). SFCT was not related to the following systemic and ocular parameters: systolic blood pressure (*P* = 0.49), presence of diabetes mellitus (*P* = 0.62), HbA1c value (*P* = 0.20), serum concentrations of high-density lipoproteins (*P* = 0.37), low-density lipoproteins (*P* = 0.053), triglycerides (*P* = 0.99), central corneal thickness (*P* = 0.98), and corneal diameter (*P* = 0.90).

### Multivariate analysis of SFCT

Multivariate analysis showed that the SFCT was significantly related to the systemic and ocular parameters including younger age (*P* < 0.001), male gender (*P* < 0.001), better best corrected visual acuity (logMAR (logarithm of minimal angle of resolution); *P* = 0.001), shorter axial length (*P* < 0.001), flatter cornea (*P* < 0.001), deeper anterior chamber (*P* < 0.001) and thicker lens (*P* < 0.001)^12^. Subsequently, we adjust the results with these parameters, and added hypertension associated parameters (diastolic blood pressure, history and years of arterial hypertension and stage of hypertensive retinopathy) in the multivariate analysis, separately from each other to the list of independent parameters. SFCT also significantly related to the stage of hypertensive retinopathy (*P* < 0.001; B: 27.64 (95% CI 23.46, 31.82); beta: 0.260) (Table 2). But not significantly associated with diastolic blood pressure (*P* = 0.94), history (*P* = 0.95) and years (*P* = 0.91) of hypertension.

**Table 2:** Associations between the subfoveal choroidal thickness and systemic and ocular parameters in the Beijing Eye Study 2011.

Parameter	*P*-value	Standardized coefficient Beta	Regression coefficient B	95% confidence interval
**Systemic parameters**				
Age (YEARS)	< 0.001	− 0.32	− 3.52	− 4.05, − 2.98
Men / women	< 0.001	− 0.13	− 27.06	− 35.67, − 18.44
**Ocular parameters**				
Axial length (mm)	< 0.001	− 0.33	− 31.37	− 36.62, − 26.12
Anterior chamber depth (mm)	0.004	0.07	21.88	6.92, 36.84
Corneal curvature radius (mm)	< 0.001	0.09	35.97	17.29, 54.66
Best corrected visual acuity (logarithm of Minimal Angle of resolution (logMAR))	< 0.001	0.11	53.48	31.32, 75.64
**Hypertension associated parameters**				
Stage of hypertensive retinopathy	< 0.001	0.26	27.64	23.46. 31.82

### Univariate analysis of hypertension or hypertensive retinopathy

In the final part of the statistical analysis, we firstly assessed relationship between hypertension or hypertensive retinopathy, with systemic and ocular parameters. We found that in our study population in a univariate analysis, hypertension was significantly associated with higher age (*P* < 0.001), urban region of habitation (*P* < 0.001), higher weight (*P* < 0.001), lower height (*P* < 0.001), higher intraocular pressure (*P* = 0.003), self report of cardiovascular disease (*P* = 0.013) and infarction (*P* = 0.022), aspirin intake (*P* < 0.001), and frequency of reported snoring (*P* = 0.011); while it not related to SFCT (*P* = 0.11), best corrected visual acuity (*P* = 0.17), axial length (*P* = 0.62), corneal curvature radius(*P* = 0.92), lens thickness (*P* = 0.59), serum concentrations of glucose (*P* = 0.07), and level of education (*P* = 0.16).

### Multivariate analysis of hypertension or hypertensive retinopathy

Multivariate analysis showed that the presence of hypertensive retinopathy was significantly associated with SFCT (*P* < 0.001), age (*P* < 0.001), absence of cardiovascular disease (*P* = 0.005), and country region of habitation (*P* < 0.001) (Table 3), while it was not significantly associated with axial length (*P* = 0.35), best corrected visual acuity (*P* = 0.44), refractive degree (*P* = 0.93), intraocular pressure (*P* = 0.58), weight (*P* = 0.72), serum concentrations of glucose (*P* = 0.07), corneal curvature radius (*P* = 0.61), level of education (*P* = 0.52), smoking (*P* = 0.56) and drinking (*P* = 0.40).

**Table 3:** Associations between the presence of hypertensive retinopathy and systemic and ocular parameters in the Beijing Eye Study 2011.

Parameter	Regression coefficient B	*P*-value	Odds ratio	95% confidence interval
**Systemic parameters**				
Age (years)	0.04	< 0.001	1.04	1.02, 1.06
Rural/urban region of habitation	− 0.85	< 0.001	0.43	0.31, 0.58
**Ocular parameters**				
SFCT	0.11	< 0.001	1.01	1.01, 1.01
**Hypertension associated parameters**				
Cardiovascular disease	− 0.47	0.005	0.62	0.45, 0.87

Multivariate regression analysis showed that the stage of hypertensive retinopathy was significantly related to SFCT (*P* < 0.001, B: 0.003 (95% CI 0.003, 0.004); beta: 0.354), age (*P* < 0.001, B: 0.017 (95% CI 0.010, 0.024); beta: 0.166), serum concentrations of glucose (*P* = 0.004, B: 0.043 (95% CI 0.014, 0.073); beta: 0.080), country region of habitation (*P* < 0.001, B: − 0.349 (95% CI − 0.473, − 0.225); beta: − 0.168), and absence of cardiovascular disease (*P* = 0.004, B − 0.191 (95%CI − 0.321, − 0.060); beta: − 0.082), while it was not significantly associated with axial length (*P* = 0.86), best corrected visual acuity (*P* = 0.71), refractive degree (*P* = 0.90), intraocular pressure (*P* = 0.97), weight (*P* = 0.89), corneal curvature radius (*P* = 0.97), level of education (*P* = 0.70), smoking (*P* = 0.92) and drinking (*P* = 0.96).

## Discussion

In the Beijing eye study 2011, SFCT was significantly related to the stage of hypertensive retinopathy (*P* < 0.001) after adjustment with age, gender, best corrected visual acuity, axial length, corneal curvature radius, anterior chamber depth and lens thickness. On the contrary, SFCT was not significantly related to diastolic blood pressure (*P* = 0.94), history (*P* = 0.95) and years (*P* = 0.91) of hypertension. The results revealed that hypertension was not related to subfoveal choroidal thickness as general disease, while hypertensive retinopathy was significantly associated with a thicker choroidal thickness.

Our findings in accord with the previous researches which show the systemic associations of a thinner SFCT include elder age, female gender, longer axial length, thinner lens, lower anterior champer,high corneal curvanture and lower best corrected visual acuity^13,18–21^. Only a few prior study evaluated choroidal thickness in hypertensive patients. Recently, Gök et al.^22^ examined 116 patients of systemic hypertension and 116 normal subjects which matched the age (*P* > 0.05) and showed that there was no significant correlation of the mean SFCT with hypertension. Masís et al.^23^ studied 112 patients with systemic hypertension and 15 healthy controls. The mean ages of the two groups were 67 and 51 years, respectively. The choroidal thickness was significantly thinner in hypertensive patients compared to controls. However, we suggest that this reflected only a between-group age difference. Similarly, the present study also showed a thinner SFCT in hypertensive patient (246.77 ± 107.22 µm vs 261.91 ± 107.73 µm, *P* < 0.001), but the normal people was significantly younger than hypertensive people (62.24 ± 9.3 vs 66.39 ± 9.8, *P* < 0.001) and after adjustment for age and other related factors, SFCT was not significantly associated with hypertension in multivariate analysis. It revealed that there was no significant difference in the SFCT between hypertensive patients and normal people.

In our study, the SFCT and level of hypertensive retinopathy had significant positive correlation (P < 0.001), after the results had been adjusted with age, gender, best corrected visual acuity, axial length, corneal curvature radius, anterior chamber depth and lens thickness. But there has been no clinical or population based research to study the relationship between the SFCT and hypertensive retinopathy. Results of related pathophysiologic study may demonstrate that positive correlation between the SFCT and the stage of hypertensive retinopathy^5,6,24^. Choroidal vasculature does not possess the autoregulatory capacity of the retinal vasculature, and hypertension could induce choroidal ischemia, which develops the lesion of choroid and retina^24^. The results have been proved by animal research, which imply that microangiopathy and visual deficits characterize the retinopathy of a spontaneously hypertensive rat model^25^. With increasing of blood pressure, acute ischemia occurred in choroids. Firstly, choroidal arterioles would constrict, leading to necrosis of the choriocapillaris and retinal pigment epithelium, chronic occlusive is characterized by extreme narrowing or occlusion of the choroidal capillaries, which may reduce the SFCT. And it was parallel to the result of our study that the SFCT of hypertensive patients without detectable hypertensive retinopathy was significantly thinner than control group (187.04 ± 78.80 µm vs 261.91 ± 107.73 µm, *P* < 0.001). If the hypertension persistent, the choroid could get into the chronic reparative phase^6^. The occluded choroidal arteries, arterioles, and choriocapillaris are recanalized, and the reparative choroidal vessel decrease in Na–K-ATPase activity^26^. . High sodium content promotes water retention with simultaneous enlargement of the choroidal thickness, and it may be confirmed by the present research, which revealed that SFCT of patients with hypertensive retinopathy was significantly thicker than hypertensive patients without hypertensive retinopathy or normal people (Table 1). Furthermore, in multivariate analysis, SFCT was significantly associated with the stage of hypertensive retinopathy, after adjustment for age, gender, best corrected visual acuity, axial length, corneal curvature radius, anterior chamber depth and lens thickness (*P* < 0.001) (Table 2). Since grade of hypertensive retinopathy was related to the choroidal thickness, one may reveal that the hypertension associated choroidal thickening was a risk factor for the development or progression of hypertensive retinopathy.

17 subjects had hypertensive choroidopathy in the study population, which showed subretinal fluid or Elschnig’s spots.The mean SFCT of the cases was 275.32 ± 78.80 µm. Although the SFCT has a downward trend, but because the sample size is too small, there is no significant difference (P = 0.23) in statistical analysis.

There are some primary limitations in our study. First of all, we should pay attention to the nonparticipation like other prevalence study. A reasonable response rate of the Beijing Eye Study 2011 was 78.8%, but, whether or not to take part in the investigation of the population could still lead to a selection bias^12,13,27,28^. Secondly, 1953 (56.3%) people fulfilled the diagnosis of hypertension in all of 3468 participants. The prevalence rate of hypertension was relatively higher in our study population is due to higher incidence of hypertension in people over age 50^1–3^. In our study population, some of the subjects have already taken antihypertensive drugs for treatment. After adjusting the results with age, gender, best corrected visual acuity, axial length, corneal curvature radius, anterior chamber depth and lens thickness, SFCT was still significantly related to the stage of hypertensive retinopathy. So we could speculate that the results and conclusions of our study were not markedly affected by the relatively higher prevalence rate of hypertension. Thirdly, a circadian (diurnal) rhythm of about 20–30 micron change in choroidal thickness measurements by OCT has been proved in previous studies by Chakraborty and others^29^. In our investigation, the participants underwent the OCT examinations at uncertain time in a day. Since we performed systemic and eye examinations in a randomized manner, but the difference of the choroidal thickness on the time of the day maybe lead to a bias in our study^30–32^. Fourthly, all participants were asked to receive detail ophthalmic examination in both eyes, including the OCT examination. Because of choroidal thickness was measured only in the right eyes, and there are still imperfections in the measurements of the left eyes, so that inter-eye differences could not be assessed^28^. Fifthly, in order to better understand the choroidal vascular changes caused by hypertension, we will investigate the effect of blood pressure on these findings of SFCT decreasing in the future.

In summary, patients with hypertensive retinopathy had an abnormal thickness of subfoveal choroid, whereas presence of hypertension was not related to subfoveal choroid thickness in a population-based study. After adjusting for age, gender, best corrected visual acuity, axial length, corneal curvature radius, anterior chamber depth and lens thickness, hypertensive retinopathy was significantly related to changes of choroidal thickness as an ocular disease, while hypertension as a systemic disease was not related to the subfoveal choroidal thickness. So one may further take into account, lesions of choroid during chronic hypertension may play an important role in development of hypertensive retinopathy.
